# Clinicopathological Studies on Vitamin D_3_ Toxicity and Therapeutic Evaluation of *Aloe vera* in Rats

**DOI:** 10.4103/0971-6580.75851

**Published:** 2011

**Authors:** Sambhaji G. Chavhan, R. S. Brar, H. S. Banga, H. S. Sandhu, S. Sodhi, P. D. Gadhave, V. R. Kothule, A. M. Kammon

**Affiliations:** Department of Veterinary Pathology India; 1 Department of Veterinary Pharmacology and Toxicology, GADVASU, Ludhiana - 141 004, Punjab, India

**Keywords:** *Aloe vera*, calcification, histopathological lesions, hypercalcemia, vitamin D_3_ toxicity

## Abstract

A study was conducted to examine the clinical signs, hematological, biochemical and histopathological changes in vitamin D_3_ toxicity at a dose rate 2 mg/kg b.wt. of vitamin D_3_ and to assess the protective effect of *Aloe vera* in vitamin D_3_ toxicity. The clinical signs observed were anorexia, progressive weight loss, difficulty in movement and respiration, diarrhea, epistaxis, subnormal body temperature and nervous signs before death. Mortality was observed in treated rats between day 10 and day 19 of treatment. The gross postmortem changes observed were severe emaciation, white chalky deposits on epicardial surface of heart, pin point white deposits on cortical surface of kidneys with pale yellow discoloration and diffused white deposits on serosal surface of stomach and intestine with bloody ingesta in lumen. The hematological changes included non-significant increase in hemoglobin and total leukocyte count and significant increase in relative neutrophil count. The biochemical changes observed were significant increase in plasma concentration of calcium, phosphorus and blood urea nitrogen, whereas a significant decrease in the concentration of albumin and total plasma protein was observed. The histopathological lesions included calcification of various organs, viz., tongue, stomach, intestines, kidney, heart, aorta, larynx, trachea, lungs, spleen, choroid plexus arteries of brain and vas deferens. The *Aloe vera* juice (2.5% in drinking water) has no protective effect on vitamin D_3_ toxicity (2 mg/kg b.wt.).

## INTRODUCTION

Nowadays, use of vitamin D_3_ (cholecalciferol) in commercial pet, livestock and infant feed supplements, multivitamin preparations and as a rodenticide has increased the risk of its toxicity. Various plant species that have a high concentration of vitamin D analogue have been reported as a source of vitamin D_3_ toxicity in livestock. The most common source of vitamin D_3_ toxicity in dogs and cats is accidental ingestion of rodenticide baits containing cholecalciferol.[1] So, the present study was designed to examine the clinical signs, hematological, biochemical and histopathological aspects and ameliorative effect of *Aloe vera* on vitamin D_3_ toxicity in rats.

## MATERIALS AND METHODS

Thirty-two healthy adult male *Wistar* albino rats were weighed and housed in laboratory animal cages. The rats were fed on standard laboratory animal feed for rat and mice, supplied by Godrej Agrovet Limited, Mumbai. The rats were kept for 7 days to acclimatize in laboratory condition prior to start of sampling protocols. On day 7, the rats were randomly divided into four groups (A, B, C and D) of eight rats in each group. Group A was given vitamin D_3_ orally at 2 mg/kg single dose daily, dissolved in groundnut oil. Group B was given vitamin D_3_ orally at 2 mg/kg dissolved in groundnut oil single dose daily and 2.5% commercial *Aloe vera* preparation in drinking water daily. Group C served as control group and was administered groundnut oil only. Group D was given 2.5% commercial *Aloe vera* preparation in drinking water daily. The rats were anesthetized by using diethyl-ether inhalation anesthesia method and blood samples were collected by retro-orbital plexus method on day 0, day 10 or just before death in 1% heparin solution for biochemistry and in ethylenediaminetetraacetic acid (EDTA) for hematological analysis. Necropsy was performed on rats died due to toxicity and relevant tissues were collected in 10% buffered formalin saline for histopathological studies. The experiment was approved by Institutional Animal Ethics Committee (IAEC), GADVASU, Ludhiana.

### Estimation of biochemical parameters

The collected blood samples were centrifuged immediately at 1500 rpm for 15 minutes and plasma was separated and stored at 4°C. These plasma samples were used for estimation of biochemical parameters. The biochemical parameters were estimated on auto analyzer by using diagnostic reagent kits (Autopak) supplied by Bayer Diagnostics India Limited, Baroda, Gujarat, India. The mean values of concentrations estimated in plasma of respective groups were compared by using one-way analysis of variance (ANOVA) test.

### Collection, processing and staining of tissue samples for histopathology

Necropsy of rats that died due to toxicity was performed as early as possible and the tissue/specimens consisting of pieces of various organs were collected in 10% formalin for histopathological examination. The control group rats were anesthetized by using diethyl-ether inhalation anesthesia method and killed by exsanguinations. The formalin-fixed tissues were washed overnight in running tap water, dehydrated in ascending grades of alcohol and cleared in benzene. The 4–5 *μ*m thick tissue sections were cut from the paraffin embedded tissues and were stained with hematoxylin and eosin stain (HandE) for routine histopathology. Wherever necessary, duplicate sections were stained to confirm calcification in tissues with Von Kossa stain.[2]

## RESULTS AND DISCUSSION

### Clinical signs observed

On day 8 of treatment, the rats of group A and group B were dull and showed decrease in feed and water intake. These signs became more intense with course of treatment. On days 10, 11 and onward, some rats of the treatment groups stopped taking feed and water. They were dull, showing difficulty in movement and rigidity of limbs. They showed ataxic movements or could not even open their mouth. The body coat was ruffled and the animals showed severe cachexia. Diarrhea was observed in rats of groups A and B besides difficulty in respiration, shivering and epistaxis. Before death, there were nervous signs like aimless running, rolling and epileptic seizures. On day 10, one rat from group A and two rats from group B were found dead. Mortality of rats in groups A and B was observed between day 10 and day 19 of treatment.

A significant decrease in body temperature of groups A and B was recorded on day 10 of treatment as compared to their average body temperature before treatment on day 0, while no significant change was found in the body temperature of rats in groups C and D [Table 1].

**Table 1:** Effect on body temperatures of treated and control group

Day	Group A (°F) (Mean±SD)	Group B (°F) (Mean±SD)	Group C(°F) (Mean±SD)	Group D (°F) (Mean±SD)
Day 0 (*n* = 8)	99.76±0.83	99.31±0.42	99.58±0.43	99.13±0.58
Day 10 (for group A *n* = 7 and for group B *n* = 6)	95.30±1.12*	95.10±0.96*	99.44±0.26**	99.06±0.57**

* Represents values differing significantly from day 0 value of that group at 5% level of significance by one-way ANOVA test;

** Represents values differing non-significantly from day 0 value of that group at 5% level of significance by one-way ANOVA test

A significant decrease in body weight of rats in groups A and B was observed on day 10 of treatment as compared to their average body weights before treatment (day 0). The total decrease in average body weight was 23.17% (76.67 g) and 22.61% (80.84 g) in groups A and B, respectively. No significant change was recorded in body weights of groups C and D [Table 2].

**Table 2:** Effect on body weight of treated and control groups

Day	Group A (g) (Mean±SD)	Group B (g) (Mean±SD)	Group C (g) (Mean±SD)	Group D (g) (Mean±SD)
Day 0 (*n* = 8)	330.83±41.03	357.50±19.42	354.28±33.09	357.85±49.23
Day 10 (for group A *n* = 7, and for group B *n* = 6)	254.16 ±33.82*	276.66 ±17.79*	357.85 ±32.25**	359.28 ±48.08**

* Represents values differing significantly from 0 day value of that group at 5% level of significance by one-way ANOVA test;

** Represents values differing non-significantly from 0 day value of that group at 5% level of significance by one-way ANOVA test

The clinical signs of vitamin D_3_ toxicity like anorexia, diarrhea, progressive emaciation/weight loss, dehydration, weakness and difficulty in movement observed in the present study are in consonance with earlier findings in different species.[3,4] Morrow[5] reported that with unregulated increase in plasma calcium and phosphorus in vitamin D_3_ toxicity, their product (calcium × phosphorus) can rise above 60, which causes mineralization of tissues/organs like kidneys, gastrointestinal tract (GIT), cardiac muscles and blood vessels, leading to structural damage with eventual decreased functional capacity. This loss of function contributes to the development of ongoing and end-stage clinical signs as well as long-term signs in animals that survive. In the present study, hypercalcemia and hyperphosphatemia along with mineralization of various organs was observed. Thus, the clinical signs observed were the sequelae of biochemical and histopathological changes due to vitamin D_3_ toxicity.

### Biochemical findings

The plasma concentrations of various biochemical parameters before treatment and on day 10 of treatment are shown in Tables 3 and 4, respectively. On day 10 of treatment, the plasma concentration of calcium, phosphorus and blood urea nitrogen (BUN) in vitamin D_3_ treated groups (A and B) was found to be significantly increased, while the plasma concentration of total plasma protein and albumin was found to be significantly decreased before death as compared to their average values in control groups [Tables 3 and 4].

**Table 3:** Effect on biochemical parameters of treated and control groups on day 0

Parameter	Groups	Mean±SD	Range
Calcium (mg/dl)	Group A** (*n* = 8)	9.68±0.27	9.2–10.0
	Group B** (*n* = 8)	9.62±0.27	9.3–10.0
	Group C (*n* = 8)	9.61±0.30	9.1–10.3
	Group D** (*n* = 8)	9.50±0.36	9.1–10.1
Phosphorus (mg/dl)	Group A**	4.55±0.31	4.1–4.9
	Group B**	4.50±0.32	4.1–5.0
	Group C	4.42±0.23	4.1–5.1
	Group D**	4.60±0.34	4.1–4.9
Albumin (g/dl)	Group A**	2.87±0.19	2.6–3.2
	Group B**	2.77±0.24	2.5–3.1
	Group C	2.63±0.41	2.0–3.2
	Group D**	2.78±0.25	2.5–3.2
Total plasma protein (g/dl)	Group A**	7.12±0.35	7–8
	Group B**	7.0±0.0	7.00
	Group C	7.0±0.0	7.00
	Group D**	7.12±0.35	7–8
BUN (mg/dl)	Group A**	22.43±3.62	18.72–27.60
	Group B**	21.74±3.42	18.14–27.86
	Group C	22.26±2.86	18.24–26.78
	Group D**	22.24±2.93	18.24–26.14

^*^Represents values differing significantly from value of control rats at 5% level by one-way ANOVA test;

** Represents values differing non-significantly from value of control rats at 5% level by one-way ANOVA test

**Table 4:** Effect on biochemical parameters of treated and control groups on day 10

Parameter	Groups	Mean±SD	Range
Calcium (mg/dl)	Group A* (*n* = 7)	13.76±1.73	10.20-16.30
	Group B* (*n* = 6)	13.30±0.95	11.70-14.40
	Group C (*n* = 8)	9.72±0.32	9.2-10.3
	Group D** (*n* = 8)	9.52±0.31	9.0-10.0
Phosphorus (mg/dl)	Group A*	5.33±0.89	4.6-6.8
	Group B*	5.36±1.032	4.4-7.20
	Group C	4.37±0.14	4.1-4.6
	Group D**	4.38±0.21	4.1-4.7
Albumin (g/dl)	Group A*	0.75±0.30	0.40-1.3
	Group B*	0.87±0.44	0.2-1.50
	Group C	2.73±0.22	2.40-3.10
	Group D**	2.82±0.29	2.4-3.30
Total plasma protein (g/dl)	Group A*	5.93±0.30	5.3-6.3
	Group B*	5.76±0.27	5.3-6.1
	Group C	7.31±0.45	7-8
	Group D**	7.37±0.51	7-8
BUN (mg/dl)	Group A*	51.54±13.09	37.31-74.86
	Group B*	51.70±10.07	37.0-68.31
	Group C	22.06±2.48	18.40-25.90
	Group D**	22.13±2.95	18.10-26.14

* Represents values differing significantly from value of control rats at 5% level by one-way ANOVA test;

** Represents values differing non-significantly from value of control rats at 5% level by one-way ANOVA test

The increase in calcium levels or hypercalcemia in cholecalciferol toxicity in the present study is in consonance with the earlier studies on vitamin D_3_ toxicity by different workers. In vitamin D_3_ toxicity, the active metabolites of cholecalciferol have been reported to increase the blood calcium (hypercalcemia) level by increased resorption/mobilization of calcium from bone, increased absorption of calcium from intestine and decreased calcium excretion by kidney. The net result is high concentration of blood calcium level (hypercalcemia) and death reported due to renal and cardiac failure.[6] The significant increase found in the plasma phosphorus levels in the present study is in accordance with findings of earlier studies on vitamin D_3_ toxicity. Morrow[5] reported that in case of acute toxicity of cholecalciferol, there is a moderate rise in serum/plasma phosphorus concentration up to 11 mg/dl and it is due to stimulation of transfer of phosphorus along with calcium from bone to plasma. The net result is mineralization of the kidney, GIT, cardiac muscles and blood vessels, causing structural damage and decrease in functional capacity of these tissues and organs. The elevated level of BUN in this study indicates renal damage. Histopathologically, hypercalcemia-induced extensive glomerular and renal tubular damage (nephrocalcinosis) was seen in the present study as reported earlier in vitamin D_3_ toxicity.[7] The increased level of BUN in the present study was in agreement with that reported earlier by various workers.[8] The significant decrease found in the levels of albumin and total plasma protein was thought to be due to loss of protein due to extensive renal damage.

### Hematological findings

On day 10, the concentration of hemoglobin and total leukocyte count of groups A and B increased non-significantly as compared to group C (control) rats. The mean relative neutrophil counts of groups A and B increased significantly from group C (control) rats on day 10, whereas counts of other cells differed non-significantly from control rats. The hemoglobin, total leukocyte count and differential leukocyte count of group D rats differed non-significantly from control rats on day 10. The relative increase in neutrophil count could be due to ongoing tissue necrosis as confirmed by histopathological observations.

### Gross postmortem changes observed

**Figure 1:**
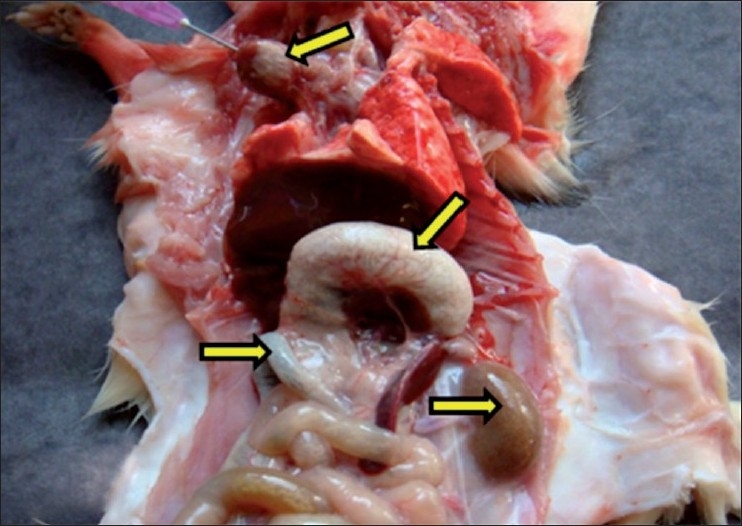
Necropsy of rats that died due to vitamin D_3_ toxicity, showing the presence of white deposits of mineralization on epicardial surface of heart, serosal surface of stomach and intestine and pin point white deposits on capsular surface of kidney. Arrows indicate sites of calcification (mineralization)

The rats died due to vitamin D_3_ toxicity (groups A and B) or sacrificed showing signs of vitamin D_3_ toxicity had severe emaciation and ruffled body coat. The heart was felt hard with white chalky deposits on epicardial surface. The stomach and intestines showed diffused white chalky deposits on serosal surface. The stomach and intestines revealed bloody ingesta in the lumen with marked hemorrhages on mucosa. Pin point white chalky deposits were observed on capsules of both kidneys [Figure 1]. The liver, lung and other organs grossly appeared normal. Gross pathological changes like bloody ingesta in stomach and intestine were observed in the present study as also reported by Long,[8] due to vitamin D_3_ toxicity in pigs.

### Histopathological findings

The following histopathological changes were observed in the tissues collected from rats died due to vitamin D_3_ toxicity (groups A and B) or sacrificed rats showing clinical signs of toxicity. The histopathological changes are presented below according to severity of lesions:

#### Tongue

**Figure 2:**
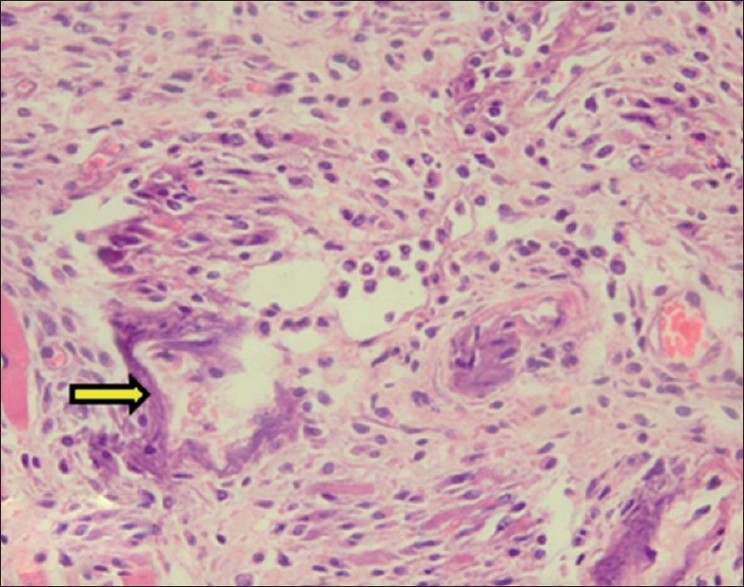
Section of tongue showing purple deposits of calcification in between muscle fibers and lymphomononuclear cell infiltration (H and E, ×150). Arrow indicate sites of calcification (mineralization)

**Figure 3:**
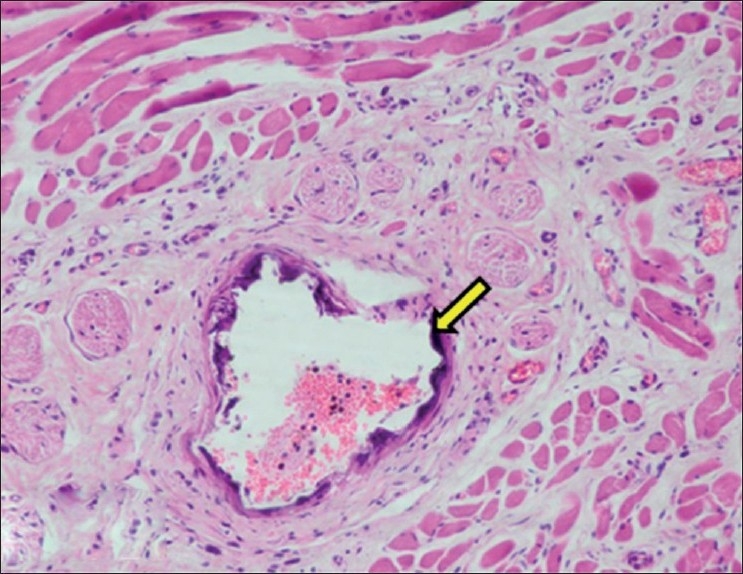
Section of tongue showing calcification within wall of blood vessel (H and E, ×150). Arrow indicate sites of calcification (mineralization)

The lamina propria, connective tissue in-between muscle fibers and tunica intima of blood vessels of tongue showed purple deposits of calcification. Around the calcified areas, there was mild lymphomononuclear cell infiltration. The calcification was also confirmed by Von Kossa staining as black deposits of calcification [Figures 2 and 3].

#### Stomach

**Figure 4:**
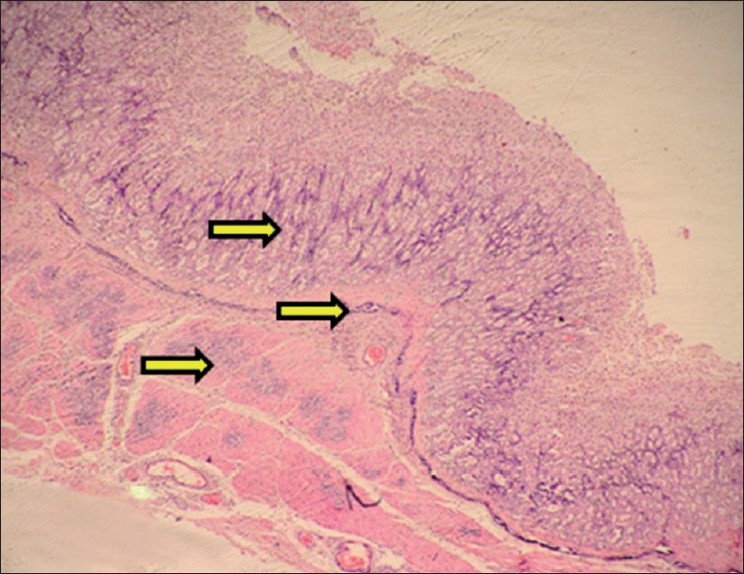
Section of stomach showing calcification of mucosa, muscularis mucosa and muscularis externa (H and E, ×75). Arrows indicate sites of calcification (mineralization)

**Figure 5:**
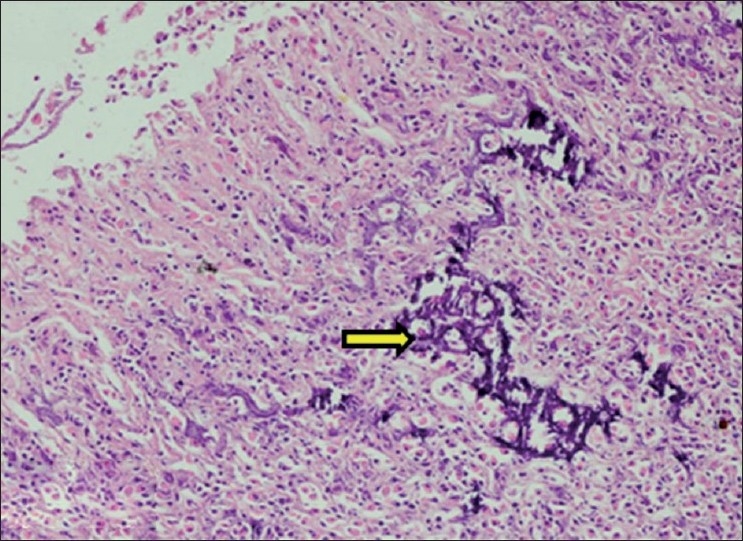
Section of stomach showing calcification within mucosa of stomach (H and E, ×150). Arrow indicate sites of calcification (mineralization)

**Figure 6:**
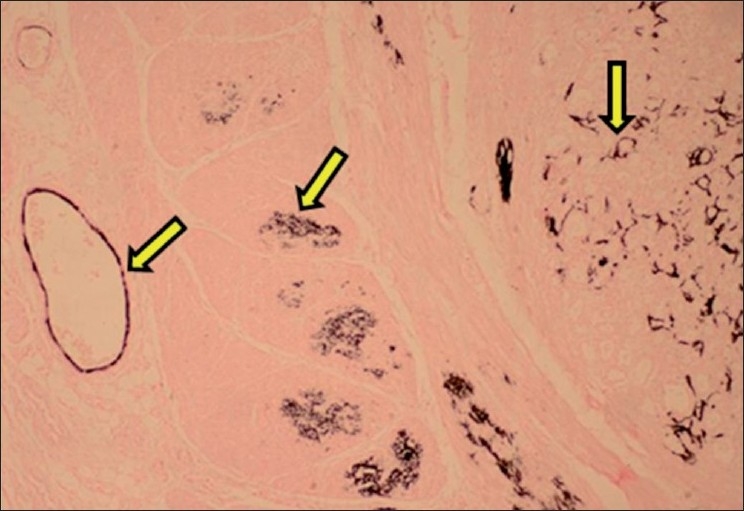
Section of stomach showing black deposits of mineralization within mucosa, muscularis mucosae, muscularis externa and within walls of blood vessels (Von Kossa stain, ×75). Arrows indicate sites of calcification (mineralization)

Calcification was observed in mucosa, muscularis mucosa and muscularis externa layers of stomach. Calcification of submucosal and serosal blood vessels was observed. Around the area of calcification, there was lymphomononuclear cell infiltration. Hemorrhagic gastritis was also observed in some rats. The calcification was confirmed by Von Kossa staining [Figures 4–6].

#### Small and large intestine

**Figure 7:**
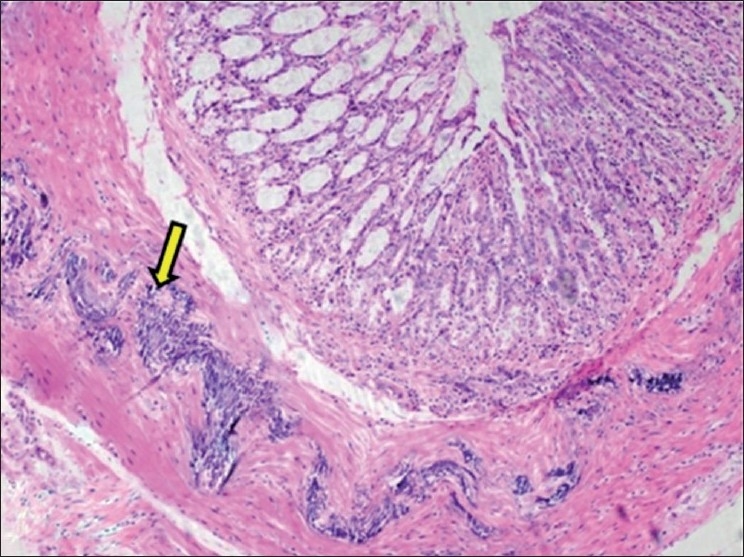
Section of large intestine showing calcification of muscularis externa layer (H and E, ×75). Arrow indicate sites of calcification (mineralization)

The lesions observed in small intestine included calcification of the Brunner’s gland epithelium and sloughing of intestinal villi epithelium. The histopathological lesions in large intestine included calcification of muscularis externa layer [Figure 7].

#### Kidney

**Figure 8:**
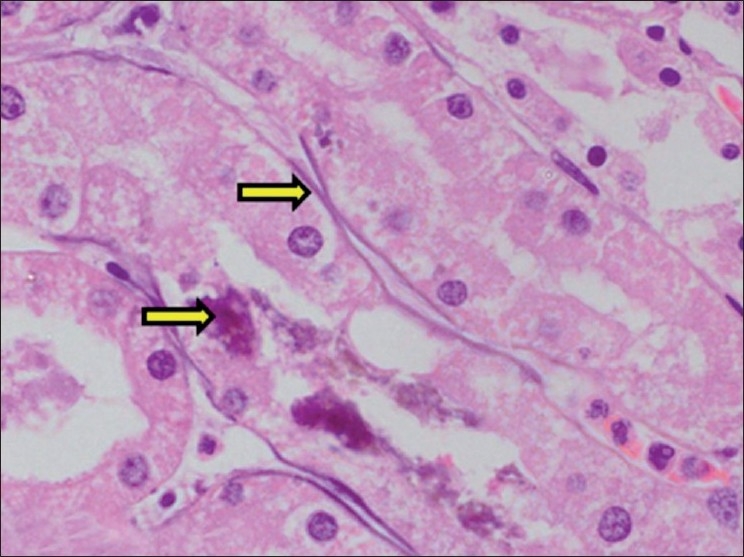
Section of kidney showing necrosis and calcification of basement membrane of tubular epithelium (H and E, ×750). Arrows indicate sites of calcification (mineralization)

**Figure 9:**
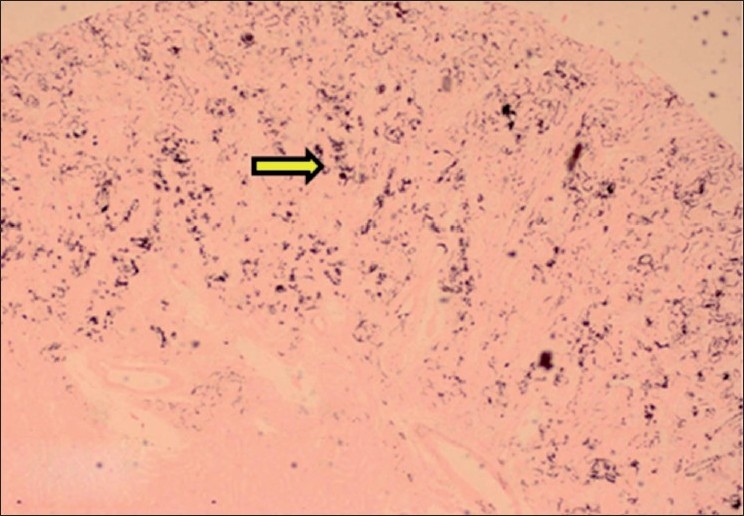
Section of kidney showing black deposits of mineralization within cortical tubules (Von Kossa stain, ×75). Arrows indicate sites of calcification (mineralization)

Mineralization (nephrocalcinosis) was observed mainly in cortex and medulla. The tubular epithelium showed calcification and coagulative necrosis along with presence of proteinaceous casts in the lumen. The calcification was also observed in basement membranes of tubules. In medulla, mineralization and degeneration was mainly observed in the collecting tubules. The walls of renal blood vessels showed calcification. Mineralization was observed in glomerular capsules and inside the glomerulus. The deposits of mineralization were also present in interstitium [Figures 8 and 9].

#### Larynx and trachea

**Figure 10:**
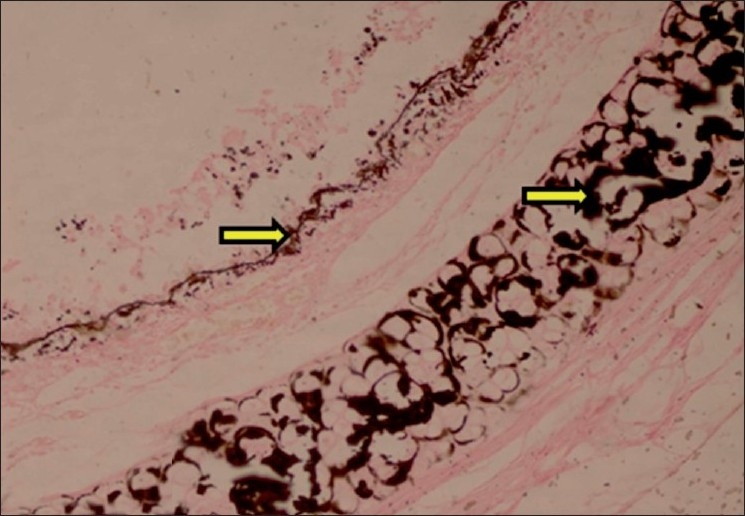
Section of trachea showing black deposits of calcification within cartilage and basement membrane of mucosal epithelium (Von Kossa stain, ×150). Arrows indicate sites of calcification (mineralization)

Calcification was observed in lamina propria of larynx. The tracheal cartilage and basement membrane of tracheal mucosal epithelium showed mineralization. The tracheal mucosa was infiltrated with inflammatory cells with exfoliated mucosal epithelial cells [Figure 10].

#### Lungs

**Figure 11:**
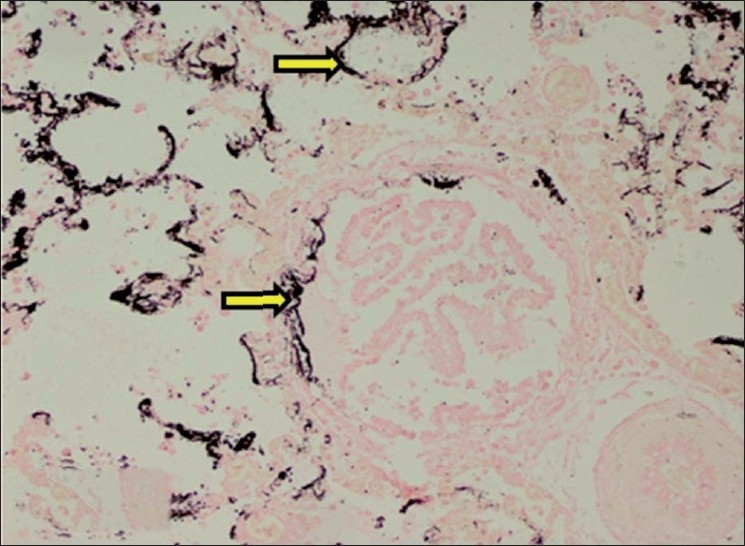
Section of lung showing black deposits of mineralization within alveolar septae and bronchial mucosa (Von Kossa stain, ×150). Arrows indicate sites of calcification (mineralization)

Calcification of bronchial mucosal epithelium was observed. In lungs, brown dirty color granular deposits of calcium were observed in alveolar and interalveolar septae. There was infiltration of lymphocytes, macrophages, occasional neutrophils and few giant cells in the thickened alveolar septae. Other histopathological changes in lungs included marked hemorrhage, emphysema, and edema. The calcification in bronchi and lung was confirmed by Von Kossa staining. The Von Kossa staining of lung sections showed extensive mineralization of alveolar septae [Figure 11].

#### Heart

**Figure 12:**
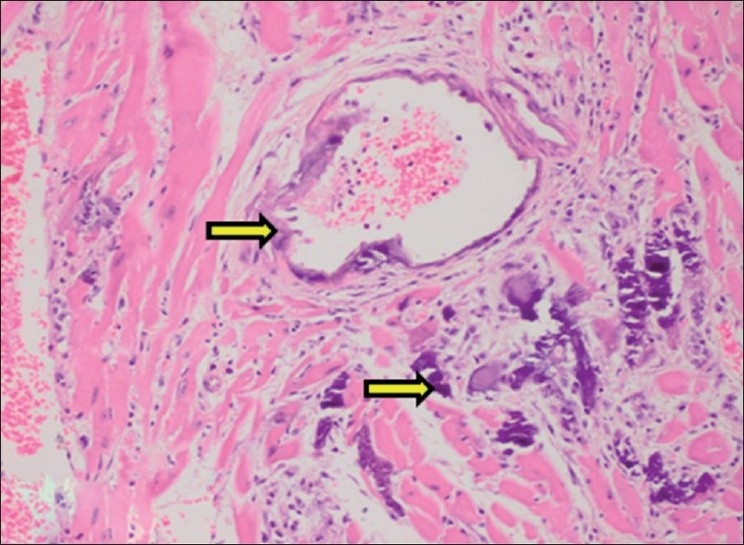
Section of heart showing marked calcification within wall of coronary blood vessel and myocardial muscle fibers (H and E, ×150). Arrows indicate sites of calcification (mineralization)

**Figure 13:**
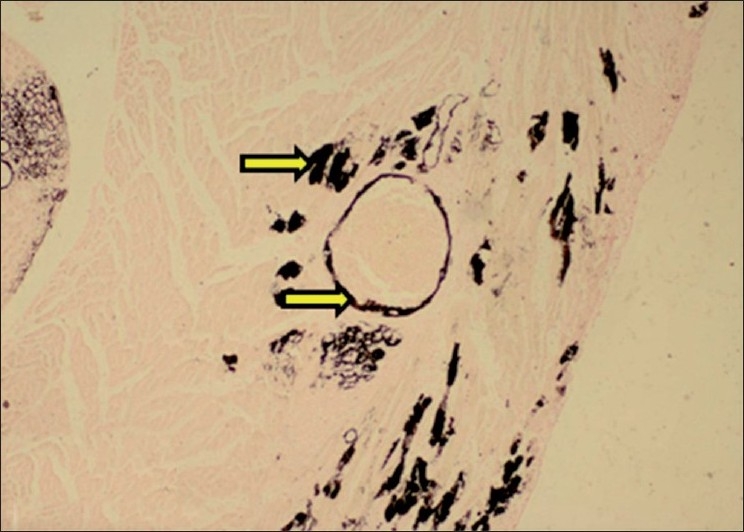
Section of heart showing black deposits of calcification within myocardial fibers and coronary blood vessel (Von Kossa stain, ×75). Arrows indicate sites of calcification (mineralization)

Calcification was observed in epicardium, myocardium and endocardium, cardiac valves and coronary arteries. The histopathological changes included calcification, degeneration and replacement of cardiac muscle cells with fibrous tissue along with lymphomononuclear cell infiltration. The epicardium and myocardium were severely affected with extensive fibrous tissue proliferation and severe lymphomononuclear infiltration [Figures 12 and 13].

#### Aorta

**Figure 14:**
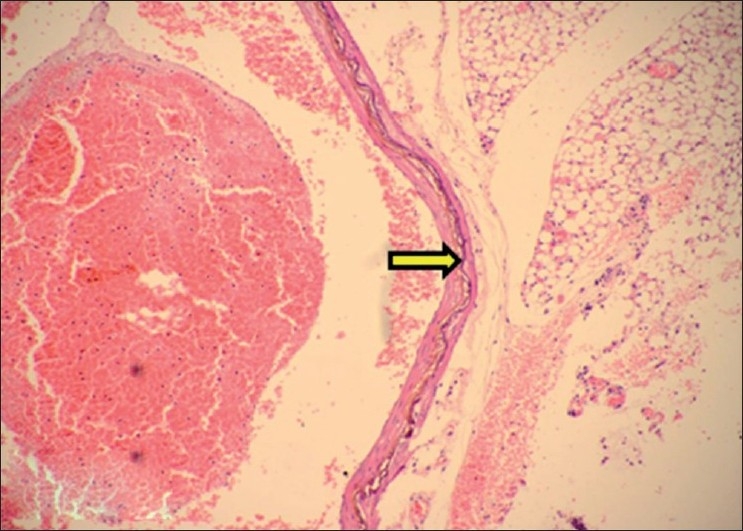
Section of aorta showing calcification within tunica media (H and E, ×75). Arrows indicate sites of calcification (mineralization)

**Figure 15:**
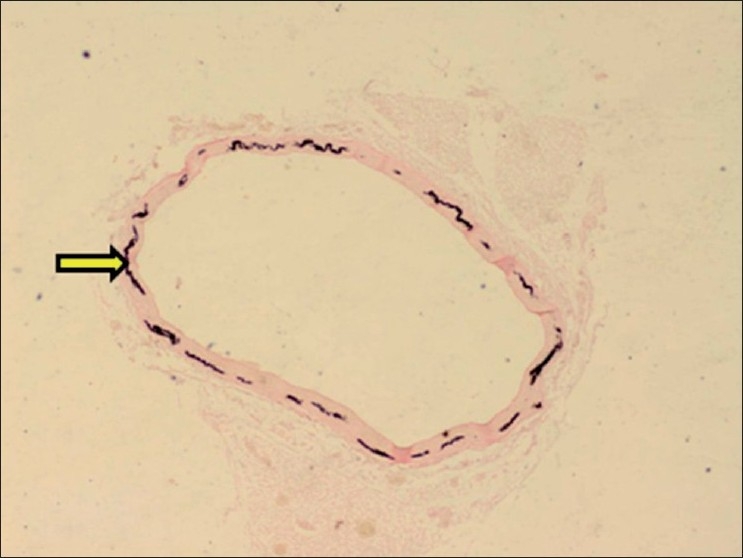
Section of aorta showing black deposits of mineralization within tunica media (Von Kossa stain, ×30). Arrows indicate sites of calcification (mineralization)

The histopathological changes observed in aorta included calcification and structural derangement of tunica media. The deposits of calcium were observed in between the elastic fibers of tunica media. The elastic fibers of tunica media were widely separated due to calcium deposits in between them causing structural derangement. The calcification in aortic media was confirmed by Von Kossa staining [Figures 14 and 15].

#### Spleen

**Figure 16:**
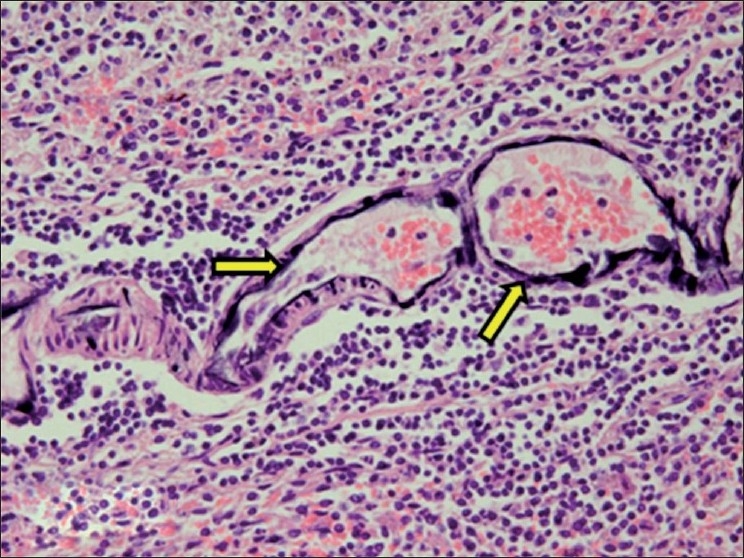
Section of spleen showing calcification of wall of central arteries of lymphatic nodules (H and E, ×300). Arrows indicate sites of calcification (mineralization)

The histopathological changes found in spleen included calcification of central arteries of lymphatic nodules and calcification of capsular surface of spleen [Figure 16].

#### Liver

The histopathological changes observed in liver included fatty/vacuolative degeneration of hepatocytes.

#### Adrenal

There was vacuolation of adrenal cortical cells and marked congestion of medulla.

#### Brain

**Figure 17:**
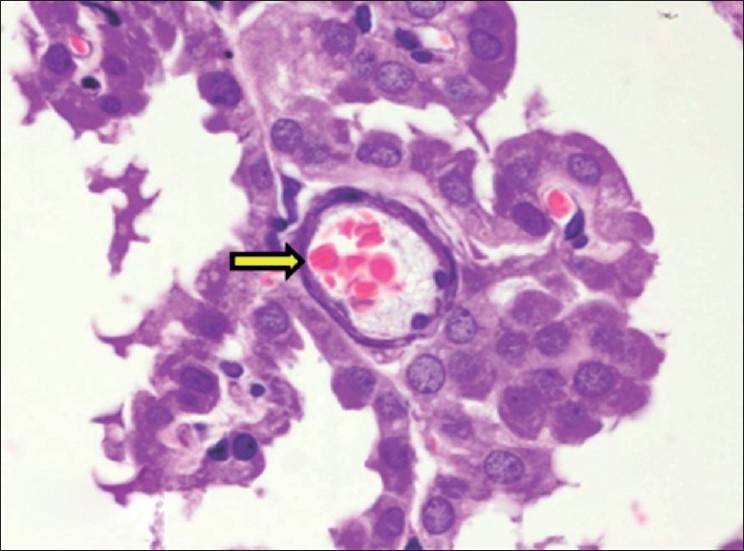
Section of cerebral hemisphere showing calcification of choroid plexus blood vessel (H and E, ×750). Arrows indicate sites of calcification (mineralization)

Calcification of choroid plexus arteries was observed along with other histopathological changes like neuronophagia and satellitosis in cerebral cortex [Figure 17]. The findings are in consonance with earlier findings reported by Morita *et al* (1995) and Hilbe *et al* (2000) in cholecalciferol toxicity. Morita *et al* (1995) and Hilbe *et al* (2000) reported the calcification of choroid plexus blood vessels in cats and dog respectively.

#### Vas deferens

**Figure 18:**
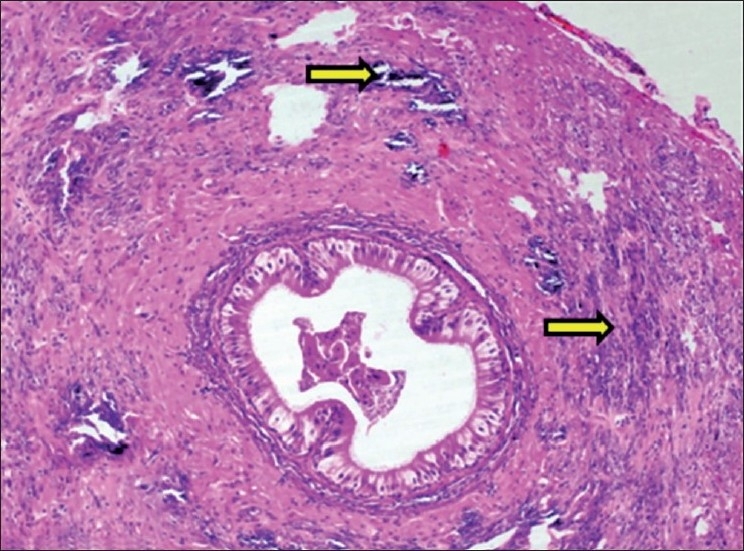
Section of vas deferens showing calcification within muscular layer (H and E, ×150). Arrows indicate sites of calcification (mineralization)

The histopathological changes observed in vas deferens included the calcification and degeneration of muscle fibers of tunica muscularis layer [Figure 18].

The effects of hypercalcemia on cells include altered cell membrane permeability, altered calcium pump activity, decreased cellular energy production and cellular necrosis. As the toxicity increases, clinical signs including hypertension, polyuria and extremely elevated serum calcium level may cause cardiac arrhythmias.[5]

The mechanism of mineralization or calcification in vitamin D_3_ toxicity is well explained by Morrow,[5] who reported that with unregulated increase in plasma calcium and phosphorus in vitamin D_3_ toxicity, their product (calcium × phosphorus) can rise above 60, which causes mineralization of tissues/organs like kidneys, GIT, cardiac muscles, blood vessels and causes structural damage that leads to decreased functional capacity of these tissues and organs. The loss of function contributes to the development of ongoing end-stage clinical signs as well as long-term signs in animals that survive. The cause of death reported in vitamin D_3_ toxicity includes cardiac and renal failure. The histopathological changes observed within various organs in present study are in agreement with earlier reports[6–8] on vitamin D_3_ toxicity in different animal species.

### Effect of *Aloe vera* on vitamin D3 toxicosis

The biochemical, hematological and histopathological parameters of group B (vitamin D_3_ at 2 mg/kg dissolved in groundnut oil daily single dose plus 2.5% *Aloe vera* juice in drinking water daily) were evaluated to assess the protective role of *Aloe vera* on vitamin D_3_ toxicity. These parameters showed same clinical signs, gross postmortem, biochemical, hematological and histopathological changes as observed in group A (vitamin D_3_ at 2 mg/kg dissolved in groundnut oil single dose daily), in comparison with control rats. The biochemical and hematological changes in group B (on day 10) differed non-significantly from group A rats.

Kawashima *et al*.,[10] reported the inhibitory effect of aspirin on vitamin D_3_ -induced hypercalcemia. Heggers *et al*.,[11] reported that *Aloe vera* gel has anti-inflammatory properties like aspirin. The earlier studies also reported the protective effect of *Aloe vera* in toxicants like lindane and arsenic.[12,13] In the present study, no protective effect of *Aloe vera* was found in vitamin D_3_ toxicity.

In conclusion, the study revealed clinical signs of severe progressive emaciation, diarrhea, difficulty in respiration and movement, nervous signs like aimless running, rolling and epileptic seizures and subnormal body temperature before death in vitamin D_3_ toxicity. The biochemical findings included increased levels of blood calcium, phosphorus and BUN, while a significant decrease was observed in the levels of total protein and albumin. The histopathological findings included calcification of various organs throughout the body. *Aloe vera* was found having no ameliorative effect against vitamin D_3_ toxicity.
